# Novel CD7-specific nanobody-based immunotoxins potently enhanced apoptosis of CD7-positive malignant cells

**DOI:** 10.18632/oncotarget.8710

**Published:** 2016-04-12

**Authors:** Jinle Tang, Jialu Li, Xuejun Zhu, Yuan Yu, Dan Chen, Lei Yuan, Zhenyang Gu, Xingding Zhang, Lin Qi, Zhishu Gong, Pengjun Jiang, Juhua Yu, Huimin Meng, Gangli An, Huyong Zheng, Lin Yang

**Affiliations:** ^1^ The Cyrus Tang Hematology Center, Soochow University, Suzhou, Collaborative Innovation Center of Hematology, Soochow University, Suzhou, China; ^2^ Suzhou Cancer Immunotherapy and Diagnosis Engineering Center, Suzhou, China; ^3^ Division of Hematology, Department of Medicine, Jiangsu Provincial Traditional Chinese Medical Hospital, Nanjing, Jiangsu Province, China; ^4^ Department of Hematology, Chinese PLA General Hospital, Beijing, China; ^5^ Beijing Key Laboratory of Pediatric Hematology Oncology, National Key Discipline of Pediatrics, Ministry of Education, Key Laboratory of Major Diseases in Children, Ministry of Education, Hematology Oncology Center, Beijing Children's Hospital, Capital Medical University, Beijing, China; ^6^ The Department of Lymphoma/Myeloma, The University of Texas MD Anderson Cancer Center, Houston, TX, USA; ^7^ The Medical Group of Zhengzhou First People's Hospital, Zhengzhou, China

**Keywords:** immunotoxin, nanobody, VHH, leukemia, target delivery drug

## Abstract

Various CD7-targeting immunotoxins have been tested for its potential in treating CD7+ malignant patients but none of those immunotoxins was approved clinically because of lacking enough efficacy and safety. Here we successfully constructed the monovalent and bivalent CD7 nanobody-based immunotoxins PG001 and PG002, both conjugated with a truncated derivative of Pseudomonas exotoxin A respectively. The prokaryotic system expressed immunotoxins not only maintained their binding specificity for CD7-positive cells with a Kd of 16.74 nM and 3.6 nM for PG001 and PG002 respectively, but also efficiently promoted antigen-restricted apoptosis of the CD7-positive leukemia cell lines Jurkat and CEM, and primary T-cell acute lymphoblastic leukemia (T-ALL) and acute myeloid leukemia (AML) cells with an *in vitro* cytotoxic activity (EC_50_) in the range of 23-30 pM for PG002. In NOD/SCID mice transplanted with CEM cells, PG001 and PG002 prevented engraftment of the cells and markedly prolonged mouse survival. Owing to the efficient antigen-restricted anti-leukemic activity of PG002, this CD7 nanobody-based immunotoxin exhibited a superior anti-CD7 positive malignancies activity than previously reported immunotoxins, and may represent a promising therapeutic strategy in treating CD7-positive leukemia and lymphoma, which still remain a significant clinical challenge.

## INTRODUCTION

T-cell lymphomas and leukemias are heterogeneous hematological malignancies. Unfortunately, conventional therapeutic strategies for aggressive adult T-cell leukemia-lymphoma (ATL) are not associated with satisfactory outcomes, mainly because ATL cells are often resistant to chemotherapeutic agents [1]. Recently, ATL treatment efforts, including allogeneic hematopoietic stem cell transplantation and molecularly targeted therapies [2, 3], have improved overall survival durations. Furthermore, treatment with the monoclonal anti-CCR4 antibody mogamulizumab produced marked cytotoxic effects on ATL cells, especially leukemic ATL cells [4].

However, more than half of ATL patients still have relapses and die with progressive disease [5, 6]. Once a patient experiences chemoresistance or a relapse, treatment options are limited, and the survival duration is short. Thus, effective novel therapies with distinctive mechanisms that can avoid multi-drug resistance and have favorable specificity and toxicity profiles are needed to treat human T-cell malignancies. Immunotoxins consisting of peptide toxins fused with tumor cell-selective antibody fragments or ligands are believed to be effective against and remain sensitive to chemoresistant T-cell disorders [7]. Recent findings indicated that immunotoxins have highly and specifically cytotoxic effects on tumor cells *in vitro* and *in vivo*, which may represent an important method of cancer therapy [7, 8].

One of the key points in using immunotoxins is to choose appropriate receptors in tumor cells. CD7 is a cell surface glycoprotein and member of the immunoglobulin superfamily [9] that is expressed on most thymocytes, peripheral blood T cells, and natural killer cells [10–12]. Many studies indicated that CD7 is expressed mainly on T-cell lymphoma and leukemia cell [13–15] but absent from at least a small portion of normal T lymphocytes [16]. Another key property of CD7 for therapeutic applications in cancer cases is its rapid internalization after binding to an antibody or antibody derivative [17], which makes it well suited for drug delivery. Accordingly, various immunotoxins specific for CD7 have been generated and tested for their anti-leukemic effects. Most studies focused on plant-derived toxins such as ricin, saporin and derivatives [18–22], in while one anti-CD7 monoclonal antibody-ricin A chain immunotoxin entered in clinical trial, resulting in two partial responses and one minimal response in eleven patients with reproducibly induced occurrence of dose-limiting vascular leak syndrome(VLS) [21]. Unfortunately, none of those immunotoxins was approved clinically because of lacking enough efficacy and safety. For examples, studies show that dgA, a ricin derived toxin, is cytotoxic to endothelial cells, which become leaky even before cytotoxicity can be measured [23]. Another toxin, a truncated form of *Pseudomonas* exotoxin A (ETA' or PE38), fused to a CD7 scFv fragment caused only approximately 20% cell death of primary leukemia-derived cells, and without further examination in *in vivo* model, implying that T-lineage leukemia cells may not be sensitive to ETA', or further improvement for the reported CD7 scFv is needed [24]. Indeed, anti-CD22 variable domain formed immunotoxin with ETA' showed impressive 46% complete remission without obvious dose-limited toxicity (DLT) in the clinical trial for hairy cell leukemia patients, suggesting ETA' is a potent toxin for at least some lymphocytes [25]. Therefore, novel anti-CD7 variable fragments may provide us a new option to improve the immunotoxin efficacy on T-cell lymphomas and leukemias.

To develop novel anti-CD7 antibody, nanobody is selected as our development strategy for those reasons: nanobody is an antibody fragment consisting of a single monomeric variable antibody domain derived from camelidae heavy-chain antibodies that was discovered by Hamers-Casterman et al. [26]. The outstanding biochemical and physical properties of nanobodies make them exceptional candidates for targeted delivery of biologically active drugs [27]. Investigators have shown that nanobodies can be coupled with toxins and other functional molecules, and then used to deliver conjugates to cancer cells for the treatment of cancer and other diseases [28–31].

In the present study, we have chosen to design nanobody-PE38 immunotoxin for two reasons: 1) nanobody should have reduced immunogenicity, because most human-anti-mouse antibody responses (HAMA) are directed against the Fc-portion of whole antibodies [32] and nanobodies are weakly immunogenic in humans [33]; 2) it has been reported that ETA-based toxins have approximately 1000-fold lower affinity for endothelial than ricin-derived toxins [34] and should therefore cause far fewer vulvar lichen sclerosus symptoms [35].

Here, we characterized two CD7 nanobody-based immunotoxins effects on T-ALL cell lines and patient-derived primary T-ALL and AML cells *in vitro*, and evaluated their anti-leukemic potential *in vivo*.

## RESULTS

### Screening and characterization of anti-CD7 nanobodies

**Figure 1:**
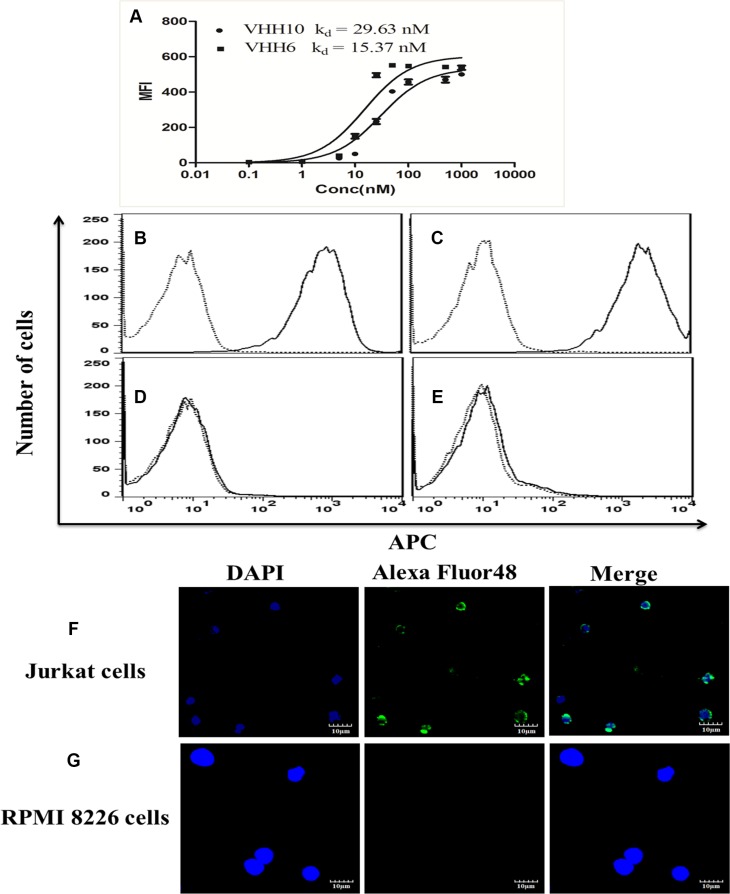
Specific binding of nanobodies to CD7-positive cells with nanomolar affinity for the cells (**A**) Binding curves for VHH6 and VHH10 and affinity (K_d_) of them for highly CD7-expressing Jurkat cells. The cells were stained with purified VHH6 and examined using flow cytometric analysis. Cells were stained with purified nanobody VHH6(solid line) or with a irrelevant nanobody (dotted line) at the same concentration and analyzed by FACS. (**B**) CD7-positive Jurkat cells. (**C**) CD7-positive CEM cells. (**D**) CD7-negative RPMI8226 cells. (**E**) CD7-negative H460 cells. Confocal fluorescence microscopic analysis of the binding of VHH6. Jurkat cell(CD7-positive; (**F**), RPMI8226 cell (CD7-negative; (**G**) cells were stained with the nanobody VHH6.

To obtain nanobodies against CD7, we screened a phage nanobody library constructed using the immunoglobulin repertoire of peripheral blood lymphocytes obtained from llamas immunized with CD7-positive Jurkat cells. We panned the phage display library with intact cells as described previously [36, 37] using parental 293T and stabilized CD7-expressed 293T cells. After four rounds of screening, 30 clones were characterized for their cell-binding ability using a whole-cell enzyme-linked immunosorbent assay approach [36]. Finally, 12 CD7-positive clones were sequenced (Supplementary Figure S1), and two clones (VHH6 and VHH10) with different complementarity determining region sequences were chosen for further analysis. VHH6 and VHH10 were subcloned into pET28a vector to induce soluble nanobody expression and purified them (Supplementary Figure S2). The affinity of VHH6 and VHH10 on jurkat cells was determined with flow cytometry as described previously [38]. Interestingly, both exhibited strong binding ability with Jurkat cells, for which VHH6 had superior affinity (15.37 nM versus 29.63 nM for VHH10) (Figure 1A). We further examined the specificity of VHH6 for CD7 using flow cytometric and immunofluorescence assays. As shown in Figure 1B and 1C, VHH6 exhibited marked binding to the CD7-positive T-cell lines Jurkat and CEM, but failed to bind the RPMI8226 and H460 cells (Figure 1D and 1E). We also incubated VHH6 with CD7 stabled line 293T cells (293T-CD7) and 293T cells. Supplementary Figure S3A and S3B showed that VHH6 specifically bound to 293T-CD7 cells but not parental 293T cells. The VHH6 also did not bind to CD7 negative cell line Ramos (Supplementary Figure S3C). Immunofluorescence experiments also indicated that VHH6 bound to Jurkat cells but not to RPMI8226 cells (Figure 1F and 1G). We also tested the specificity of VHH6 for H460 cells transiently transfected with pcDNA3.1-CD7. VHH6 strongly bound to pcDNA3.1-CD7 transfected H460 cells, but not to pcDNA3.1-transfected control cells (Supplementary Figure S4A and S4B). Importantly, immunofluorescence results from H460 cells were highly similar to flow cytometry results for H460 cells using commercially available antibody (Supplementary Figure S4C and S4D). We used the VHH6 and commercially available antibody to test the CD7 expression in transfected H460 cells by flow cytometry. The result indicated that the VHH6 and commercially available antibody showed similar binding ability (Supplementary Figure S4E). Thus, the above data suggests that VHH6 is a high-affinity, highly specific nanobody.

### Production and characterization of monovalent nanobody-based immunotoxin PG001

**Figure 2:**
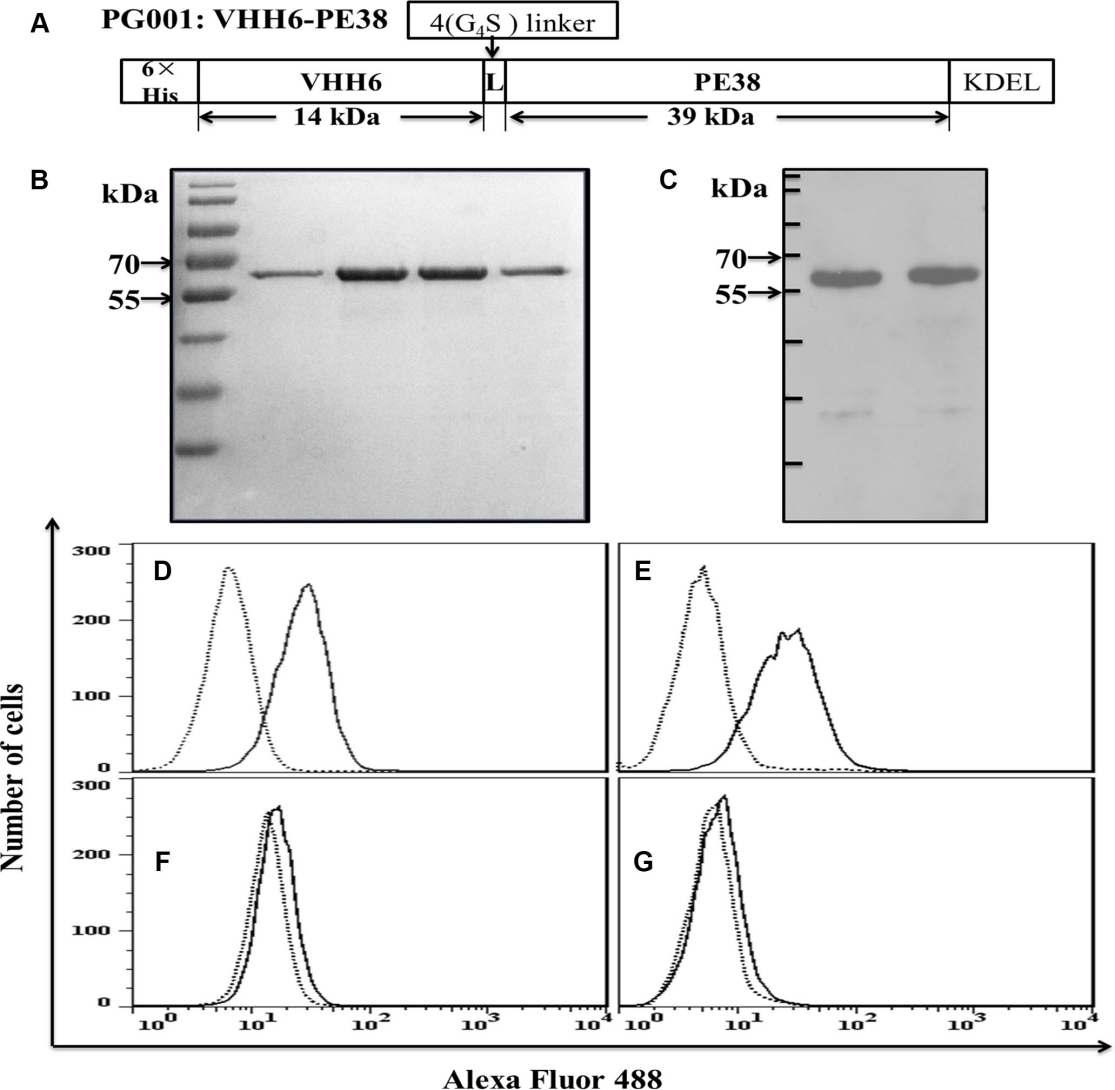
Design and characterization of the recombinant immunotoxin PG001 (**A**) Design of PG001. 6 × His, hexahistidine tag; VHH6, CD7-specific nanobody; 4 (G_4_S), flexible linkers consisting of glycine and serine residues; ETA fragment consisting of domains II and III of the *PE*; KDEL, endoplasmic reticulum retention motif. The molecular masses of the fragments in kDa were calculated from their amino acid sequences. (**B**) purity of the purified recombinant PG001 eluted from a nickel column and evaluated using Coomassie brilliant blue staining. Lanes 1–4, elution fractions 1–4. (**C**) Western blot of the recombinant PG001 using an anti-His-antibody. Cells were stained with purified PG001 (solid line) or a irrelevant immunotoxin (dotted line) and analyzed usig flow cytometry. (**D**) CD7-positive Jurkat cells. (**E**) CD7-positive CEM cells. (**F**) CD7-negative RPMI8226 cells. (**G**) CD7-negative H460 cells. The data are representative of three separate experiments.

A schematic of the monovalent immunotoxin PG001 is shown in Figure 2A. Purified PG001 exhibited greater than 90% purity, with a yield of about 10 mg from 1L of a bacterial culture. A single affinity purification cycle resulted in a highly enriched protein reactive with an antibody specific for the 6 × His tag (Figure 2B and 2C). The recombinant immunotoxin retained specific binding to CD7-positive Jurkat and CEM cells (Figure 2D and 2E), but did not bind to CD7-negative RPMI8226 or H460 cells (Figure 2F and 2G), indicating that PG001 is a CD7-specific nanobody-based immunotoxin.

### Dose-dependent, antigen-specific induction of apoptosis by PG001

**Figure 3:**
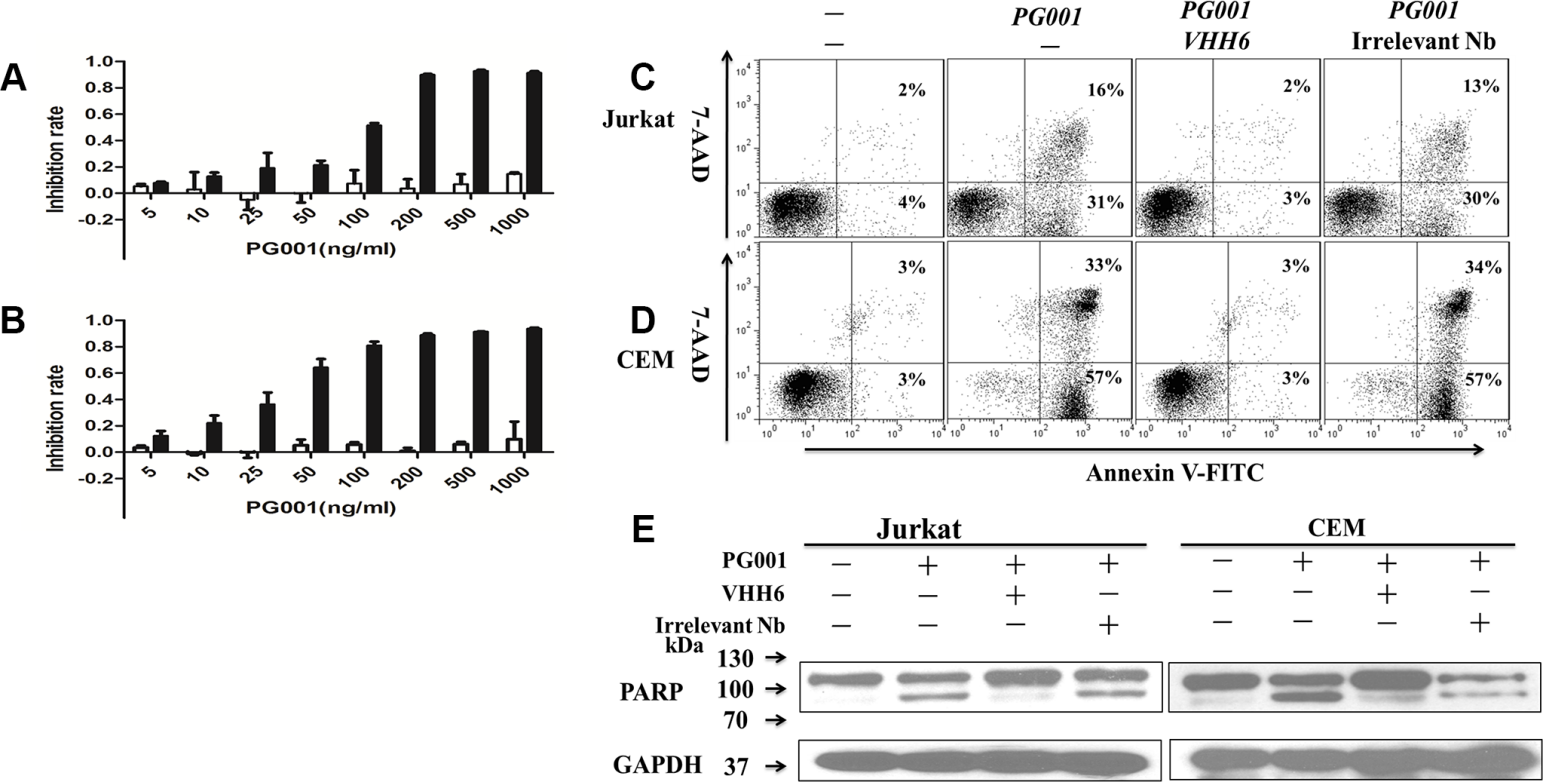
Specific induction of leukemic T-cell growth inhibition by PG001 via apoptosis in a dose-dependent manner (**A**) and (**B**) CD7-positive Jurkat cells(black bars) and CD7-negative RPMI8226 cells (white bars; A) as well as CD7-positive CEM cells (black bars) and CD7-negative H460 cells (white bars; B) were treated with PG001 at different concentrations for 72 hours. Cell-growth inhibition was measured using a WST-8 assay. The bars represent the mean values from three independent experiments. Standard deviations are indicated by the error bars. The cytotoxicity of PG001 was blocked by co-incubation with a 100-fold molar excess of parental nanobody. (**C**) Jurkat and (**D**) CEM cells were treated with a single dose of 150 ng/mL PG001 alone or in the presence of a 100-fold molar excess of the parental nanobody VHH6 and irrelevant nanobody. Forty-eight hours later, the cells were stained with annexin V and 7-AAD. The percentage of cells undergoing early apoptosis (annexin V-positive and 7-AAD-negative) is shown in the bottom right quadrant of each plot. The percentage of dead cells (annexin V- and 7-AAD-positive) shown in the upper right quadrant of each plot. (**E**) Detection of cleaved PARP confirmed antigen-specific induction of apoptosis in Jurkat and CEM cells by PG001. Data show one representative experiment out of three performed.

PG001 cytotoxic activity was determined using WST-8 assay. The results indicated that PG001 markedly suppressed Jurkat and CEM cell growth in a dose-dependent manner at low nanomolar concentrations (EC_50_, 1.0 nM for Jurkat cells and 0.5 nM for CEM cells) (Figure 3A and 3B). But the agent failed to inhibit CD7 negative RPMI8226 and H460 cells proliferation (Figure 3A and 3B). Meanwhile, the VHH6 nanobody, PE38, and irrelevant monovalent immunotoxin has no obvious toxicity on Jurkat and CEM cells, respectively (Supplementary Figure S5). To determine whether cellular growth inhibition is induced via apoptosis, we examined cells by annexin V and 7-AAD staining. Annexin V-positive, 7-AAD-negative early apoptotic cells were clearly detectable among Jurkat and CEM cells treated with PG001 for 24, 48, and 72 hours at 150 ng/mL (Supplementary Figure S6). We also observed that PG001 induced cell-growth inhibition via apoptosis in a time-dependent manner but did not suppress RPMI8226 cells cell growth (Supplementary Figure S6). The cytotoxic effect of PG001 was blocked by co-incubation of the cells with a 100-fold molar excess of the parental nanobody, but irrelevant nanobody failed to block the cytotoxicity (Figure 3C and 3D). In addition, treatment of Jurkat and CEM cells with PG001 for 24 hours induced cleavage of PARP, a characteristic feature of apoptotic cells (Figure 3E). Induction of apoptosis was also prevented by the presence of the parental nanobody (Figure 3E). However, treatment of the CD7-negative cell lines RPMI8226 and H460 did not induce cleavage of PARP (Supplementary Figure S7), demonstrating that PG001 induces apoptosis of CD7-positive leukemia-derived cell lines in an antigen-specific manner and is effective at low nanomolar concentrations.

### Bivalent nanobody-based immunotoxin PG002 and its cytotoxicity on leukemic cells

**Figure 4:**
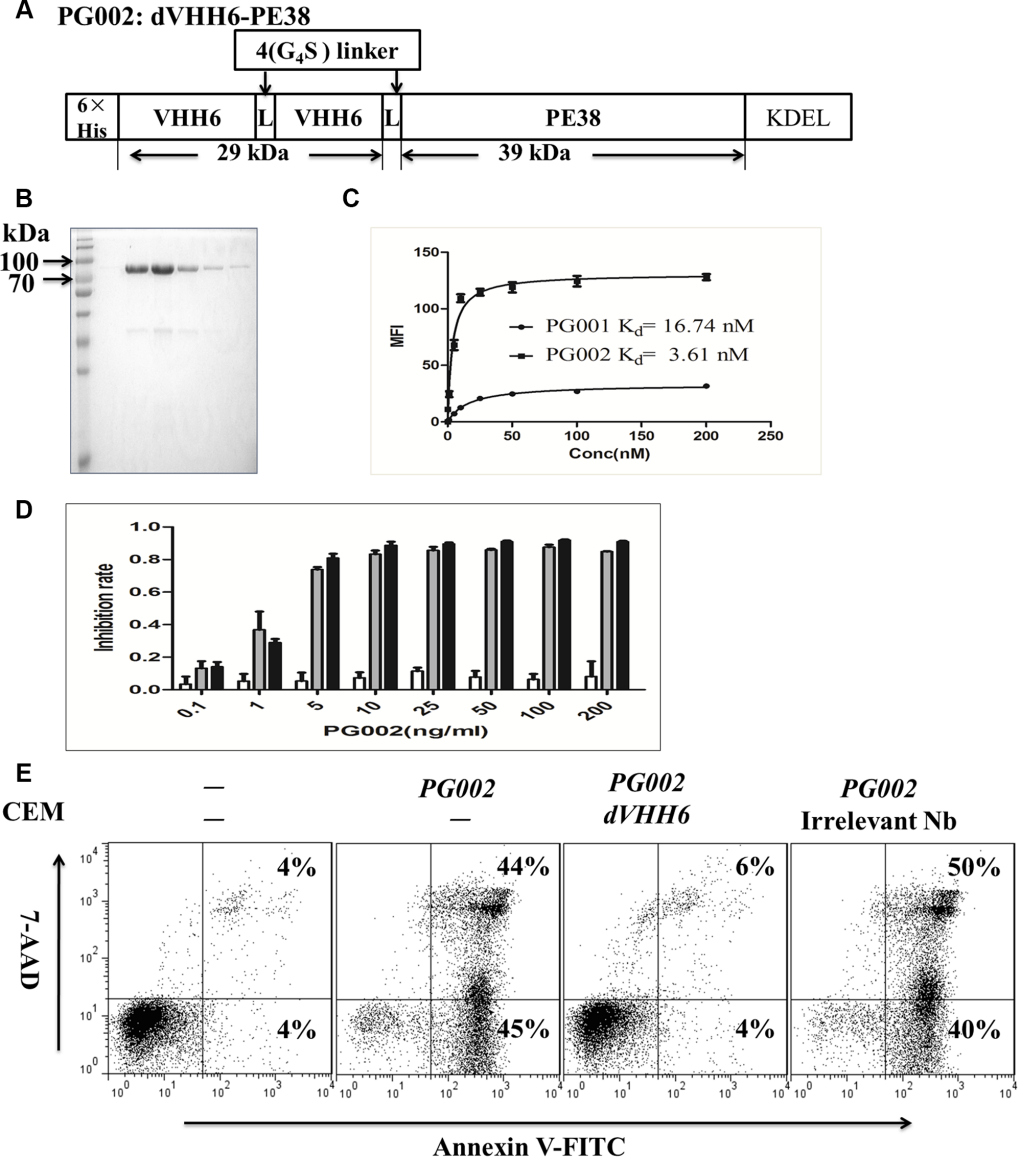
Generation of the bivalent nanobody-based immunotoxin PG002 and its cytotoxicity (**A**) Construction of PG002. VHH6, CD7-specific nanobody; 4(G_4_S), flexible linkers consisting of glycine and serine residues. (**B**) purity of the purified recombinant immunotoxin PG002 eluted from a nickel column and evaluated using Coomassie brilliant blue staining. Lanes 1–6, elution fractions 1–6. (**C**) Binding curves for PG001 and PG002 and affinity (K_d_) of them for highly CD7-expressing Jurkat cells. (**D**) CD7-positive Jurkat cells (black bars) and CD7-positive CEM cells (gray bars) as well as CD7-negative RPMI8226 cells (white bars) were treated with PG002 at different concentrations for 72 hours. Cell-growth inhibition was measured using a WST-8 assay. The bars represent mean values from three independent experiments. Standard deviations are indicated by the error bars. (**E**) CD7-positive CEM cells were treated with a single dose of 50 ng/mL PG002 alone or in the presence of a 100-fold molar excess of the parental bivalent nanobody dVHH6 and irrelevant bivalent nanobody dVHH22. Forty-eight hours later, the cells were stained with annexin V and 7-AAD. The percentage of cells undergoing early apoptosis (annexin V-positive and 7-AAD-negative) is shown in the bottom right quadrant of each plot. The percentage of dead cells (annexin V- and 7-AAD-positive) shown in the upper right quadrant of each plot.

Although PG001 shows potential in clinical applications for leukemia therapy, due to its smaller molecular weight, PG001 may have a shorter half-life than scFv-based immunotoxins *in vivo*. To improve the cell-binding affinity and *in vivo* half-life of PG001, as well as to potently induce leukemia cell apoptosis, construction of a bivalent nanobody-based immunotoxin with a longer half-life and greater cell-binding affinity is necessary. As shown in Figure 4A and 4B, the highly purified bivalent nanobody immunotoxin PG002 was obtained. Importantly, we are able to harvest about 5 mg of purified active PG002 from 1 L of a bacterial culture. We used the size exclusion chromatography to test whether the immunotoxins of PG001 and PG002 are the monomeric forms. As showed in Supplementary Figure S8, the results demonstrate that VHH6, PG001 and PG002 presented as monomers. In addition, PG002 exhibited stronger binding ability than PG001 did for CD7 positive Jurkat cells, while there was no binding to CD7 negative H460 cells (Supplementary Figure S9). This bivalent immunotoxin also maintained specific binding feature to CD7-positive cells. The affinity of PG001 and PG002 on Jurkat cells was determined by flow cytometry as described above. The result shows that the bivalent isoform PG002 (Kd = 3.61 nM, Figure 4C) has the more binding affinity than the monovalent immunotoxin PG001 (Kd = 16.74 nM, Figure 4C). Then the cytotoxic activity of PG002 was measured by WST-8 assay. The results demonstrated that PG002 significantly suppressed Jurkat and CEM cell proliferation in a dose-dependent manner (EC_50_, 30 pM for Jurkat cells and 23 pM for CEM cells) (Figure 4D). Meanwhile, PG002 did not inhibit the proliferation of RPMI8226 cells. The bivalent nanobody dVHH6 and immuntoxin dVHH22-PE38 did not suppress Jurkat and CEM growth (Supplementary Figure S10). The PG002 also markedly inhibited 293T-CD7 cell growth but without obvious toxicity on 293T cells (Supplementary Figure S11). Importantly, PG002 significantly induced CEM cell apoptosis at 50 ng/mL, and its cytotoxic effect on CEM cells was completely blocked by co-incubation of the cells with a 100-fold molar excess of the parental antibody dVHH6, but not with irrelevant bivalent nanobody (Figure 4E). Similar results were observed using Jurkat cells (data not shown), suggesting that PG002 is more effective than PG001 *in vitro*.

### Efficient induction of apoptosis in primary AML and T-ALL cells by PG001 and PG002

**Figure 5:**
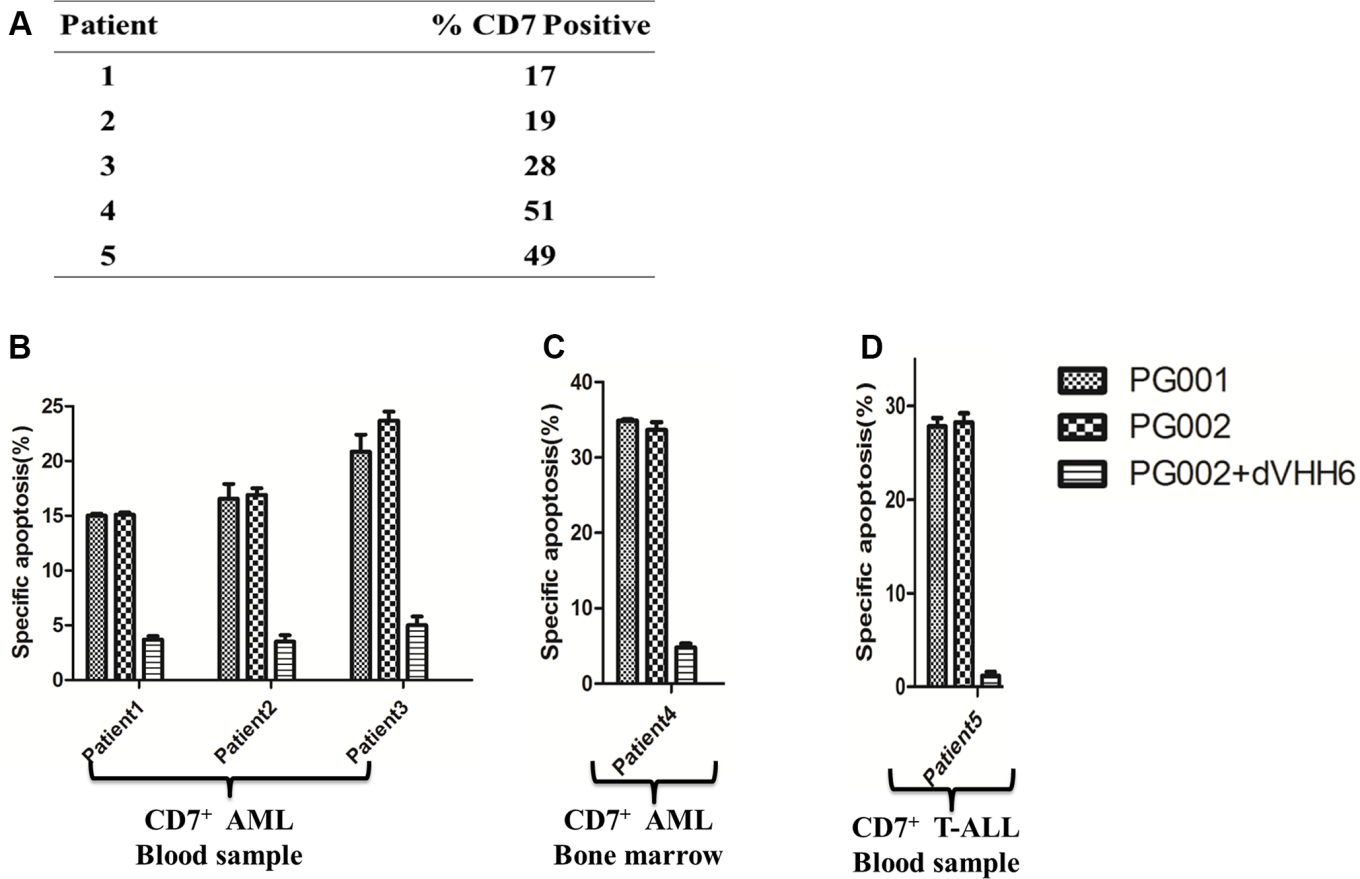
Apoptosis induction by PG001 and PG002 in patient-derived AML and T-ALL cells (**A**) Percentages of CD7-positive cells obtained from different patients. (**B**, **C** and **D**), PG002 induces apoptosis in cells derived patient. Mononuclear cells derived from peripheral blood samples obtained from three patients with CD7-positive AML (B), a bone marrow sample obtained from one patient with CD7-positive AML (C), and a peripheral blood sample obtained from one patient with T-ALL (D) were treated with 500 ng/mL PG001 or 100 ng/mL PG002 alone or in the presence of a 100-fold molar excess of the parental nanobody dVHH6. After 72 hours, cells were stained with annexin V and 7-AAD. Samples run in triplicate were analyzed, and standard deviations are indicated by the error bars.

To evaluate the cytotoxic potential of PG001 and PG002 in patient-derived primary leukemia cells, mononuclear cells obtained from five patients were treated with 500 ng/mL PG001 and 100 ng/mL PG002 respectively, or in the presence of a 100-fold molar excess of the parental nanobody dVHH6. The expression of CD7 on AML (patients 1–4) and T-ALL (patient 5) cells is shown in Figure 5A. PG001 and PG002 effectively induced apoptosis of these primary leukemia cells (Figure 5B, 5C and 5D). PG002 also induced cytotoxicity in an antigen-dependent manner, as the apoptosis in the tested samples with PG002 was blocked by the presence of the parental nanobody.

### *In vivo* effects of PG001 and PG002 in NOD/SCID mice xenotransplants

**Figure 6:**
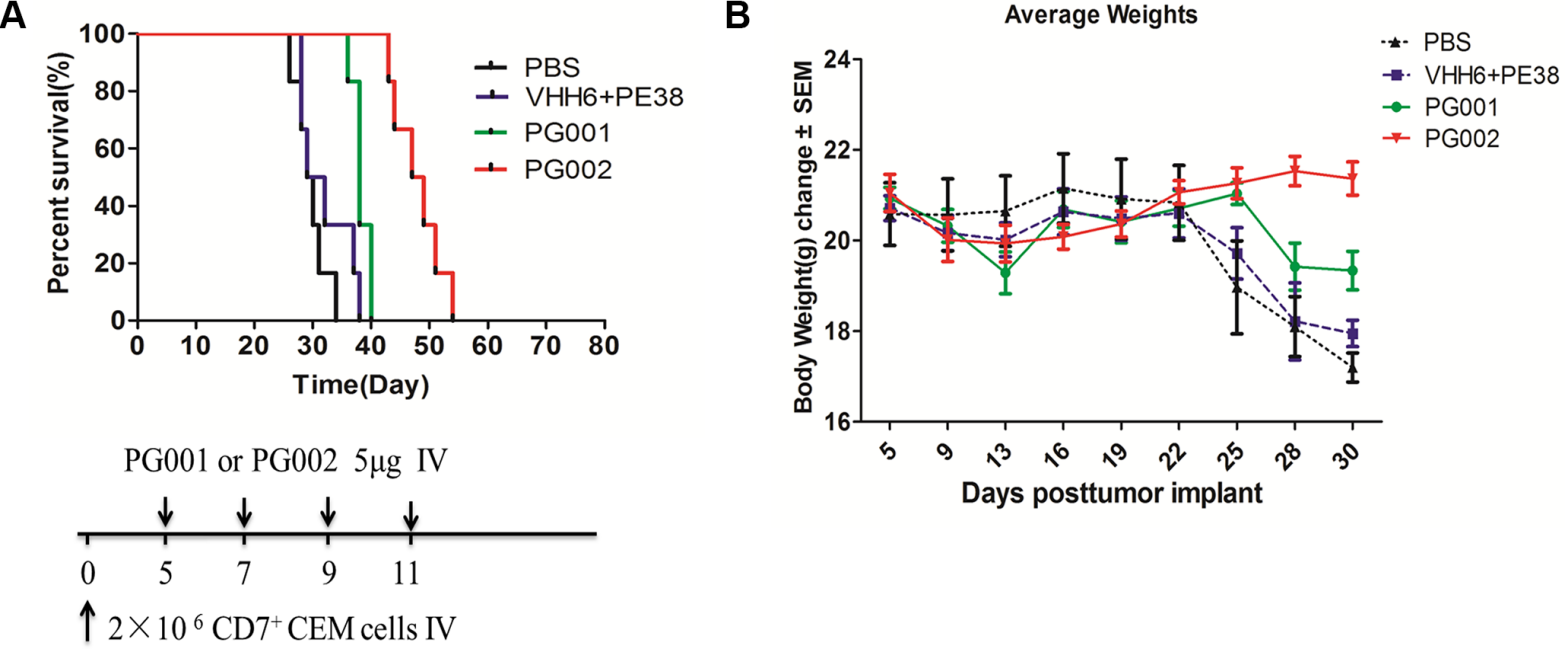
Prevention of engraftment of CEM cells in NOD/SCID mice by PG001 and PG002 (**A**) Mice received 2 × 10^6^ CEM cells intravenously to induce systemic disease. In four separate experiments, mice were given intravenous injections of 5 μg of PG002 (*n* = 6), 5 μg of PG001 (*n* = 6), 1.5 μg of free VHH6 plus 3.5 μg of free PE38 (*n* = 6), or PBS (*n* = 6). The schedule of CEM cell and drug injection is shown in the schematic below the survival curve. The vertical arrows indicate the days on which the CEM cells and immunotoxins were injected. Statistical analysis was performed using the log-rank test. (**B**) body weights of the mice in a measured every 2–3 days. The values are Presented as the mean ± standard error of the mean.

To determine the anti-leukemic potential of PG001 and PG002 in an animal model, we evaluated them in NOD/SCID mice xenotransplanted with CEM cells. We injected 2 × 10^6^ cells via the tail vein on day 0. Five days later, the mice received four intravenous injections of 5 μg of PG001 and PG002 or a noncovalent nanobody mixed with PE38 (VHH6 and PE38) in PBS. Figure 6A shows that mice given the four injections of PG001 (*P* = 0.0133) and PG002 (*P* = 0.0005) survived significantly longer than did control mice on the same injection schedule of a noncovalent nanobody mixed with PE38. Untreated controls exhibited a similar effect on survival as that in mice given the noncovalent nanobody mixed with PE38, and we did not observe a statistically significant difference between the noncovalent nanobody/PE38 and untreated control groups. The median survival durations were 29.5 days for PBS control group, 30.5 days for noncovalent nanobody/ETA group, 38 days for the PG001treated group, and 48 days for PG002 treated group. Notably, both PG001 and PG002 induced slight weight loss, which completely recovered after immunotoxin administration (Figure 6B).

We killed moribund mice and detected their leukemia cells using flow cytometric analysis with cell suspensions from spleens and bone marrows using an APC- labeled anti-CD45 antibody and PE-labeled anti-CD7 antibody. Human leukemia cells in the spleen and bone marrow samples from PG002-treated mice were detected (Supplementary Figure S12). We also detected CEM cells in other groups (data not shown).

## DISCUSSION

We identified a nanobody against CD7, VHH6, with high affinity and specificity, and then constructed the monovalent and bivalent VHH6-based immunotoxins PG001 and PG002 respectively. These two immunotoxins induced efficient antigen-specific apoptosis of T-ALL cell lines at nanomolar (PG001) and picomolar (PG002) concentrations, and effectively eliminated patient-derived primary T-ALL and AML cells via apoptosis. PG001, markedly inhibited engraftment of CEM cells in a NOD/SCID mouse model. Moreover, PG002 was much more effective than PG001 (*P* = 0.0008).

CD7 is an ideal therapeutic target for two reasons: 1) there is a subset of CD7-negative peripheral normal T cells existed [16], which may maintain immune functions required for the prevention of opportunistic infections after the generated immunotoxin eliminates all CD7-positive cells. 2) CD7 is rapidly internalized after antibody binding [17], which makes CD7 antibody a potential targeted delivery approach for patients with T-cell diseases [18–21, 24].

Traditional antibody-conjugates preparation (including scFv) is expensive with poor stability. In contrast, nanobodies are only one domain proteins, which enables them to penetrate tissues, pass through barriers such as the blood-brain barrier, and be efficiently expressed as soluble, non-aggregating recombinant proteins with high yields, and weak immunogenicity [39]. Therefore, fusion of toxins to nanobodies is a promising therapeutic strategy for cancer [29]. Additionally, the truncated PE38 of PE was therapeutically effective in patients with relapsed, chemotherapy-refractory leukemia or other hematological malignancies [35, 40, 41].

The nanobody-based immunotoxins PG001 and PG002 lack Fc fragments, resulting in reduced bindings with Fc-receptors on non-leukemia cells, similar to single-chain variable fragments immunotoxins [24, 42, 43]. When leukemia cells treated with immunotoxins were stained by annexin V and 7-AAD, and measured cleavage of PARP, the results indicated that cellular growth was inhibited via apoptosis similar as previous studies [24, 42].

The present study offered several new findings. 1) PG001 and PG002 were efficiently expressed as soluble recombinant proteins with high purity and yields from a prokaryotic expression system. The output of purified protein was about 10 mg for PG001 and 5 mg for PG002 from 1L of bacterial culture, more than 50 times greater than the output of CD7 scFv-PE38 and CD33 scFv-PE38 immunotoxins constructed by others [24, 44]. Since the cost of PG001 and PG002 production was greatly reduced by using prokaryotic expression system, make them promising in clinical applications. 2) The EC_50_ of PG002 was at the picomolar level on Jurkat and CEM cell lines, and the killing effect of PG002 was further enhanced about 30 times greater than that of PG001, similar to other immunotoxins applied in the clinic [35, 45]. The greater killing effect of PG002 *in vitro* was correlated to enhanced affinity of this immunotoxins [46]. Furthermore, PG002 increased apoptosis of freshly collected patient-derived T-ALL and AML cells *in vitro* at a single dose as low as 100 ng/mL. In contrast, previously reported CD7 scFv- PE38 immunotoxin has limited killing effect on T-ALL sample at 100 ng/ml even working for 96 hours [24]. 3) We examined the anti-leukemic potential of PG001 and PG002 in NOD/SCID mice xenotransplanted with CEM cells. The results indicated that both immunotoxins prevented engraftment of the cells and markedly prolonged survival of the treated mice. Notably, this is the first report for CD7 specific nanobody based-immunotoxins *in vivo*. In previous study, CD7 scFv-PE38 immunotoxin was only examined*in vitro* with limited inhibition effect on primary leukemia cells and there was no *in vivo* examination tested [24]. Thus, our current results have inspired us to initiate clinical trials, especially for PG002 in the near future.

We have noticed that some research teams constructed the scFvCD7:sTRAIL [47] and scFvCD7:sFasL [48] humanized immunotoxins, which effectively suppressed proliferation of CD7 positive cell lines and patient-derived CD7 positive cells. Since these immunotoxins were developed from endogenous protein of human origin, they may have less immunogenicity or nonspecific toxicity leading to vascular leak syndrome. Although these immunotoxins were not examined *in vivo* yet, we are still triggered to develop CD7 nanobody based immunotoxins with sTRAIL and sFasL in the future.

Other research groups demonstrated that bispecific single-chain immunotoxins were more effective than monospecific or bivalent immunotoxins *in vitro* and *in vivo* [49, 50]. Nanobodies can be easily formatted to construct bispecific immunotoxins to bind two different epitopes or antigens on cancer cells. Also, bispecific immunotoxins generated with nanobodies may have a nice property that is easily expressed in a prokaryotic expression system like our bivalent immunotoxin PG002. Thus, our research results laid a reasonable foundation for the further construction of bispecific immunotoxins.

In summary, we constructed monovalent (PG001) and bivalent (PG002) CD7 nanobody-based immunotoxins which can effectively eliminate CD7-positive T-ALL and fresh patient-derived T-ALL and AML cells with antigen-specificity at low concentrations. Based on the observations that PG001 and PG002 effectively and selectively killed human leukemia cells and significantly prolonged the survival of treated mice, PG001 and PG002 may deserve further evaluation of their potential for anti-leukemia in preclinical and clinical studies.

## MATERIALS AND METHODS

### Bacterial strains and plasmids

*Escherichia coli* XL1-Blue (Stratagene) was used for plasmids transformation and screening of nanobody libraries. *E. coli* BL21 (DE3; Novagen,) was used for nanobodies and immunotoxins expression. Nanobody libraries were generated using the phagemid vector pComb3XSS (Biovector NTCC), and the vector pET28a (Novagen) was used for inducible expression of soluble nanobodies and immunotoxins. The vector pcDNA3.1/myc-His B (Life Technologies) was used for construction of the plasmid pcDNA3.1-CD7, which was transfected into H460 human lung cells with lipofectamine 2000 (Life Technologies). The lentiviral vector pCDH-CMV-MCS-EF1-copGFP (System Biosciences) was used for construction of stable 293T-CD7 cell lines.

### Patient samples and cell lines

Heparinized peripheral blood and bone marrow samples were obtained from AML and T-ALL patients after they signed informed consent to participate in the study and with the approval of the Ethics Committees of Soochow University. Peripheral blood mononuclear cells were isolated using Ficoll-Paque PLUS (GE Healthcare Life Sciences) and cultured in RPMI-1640 medium (Thermo Scientific) containing 20% fetal bovine serum (Biowest). The human T-ALL cell lines Jurkat and CEM, human lung cell line H460 and human B cell line Ramos cells were cultured in RPMI-1640 medium containing 10% fetal bovine serum. The human myeloma cell line RPMI8226 were maintained in Iscove's modified Dulbecco's medium (Thermo Scientific) containing 10% fetal bovine serum and human embryonyc kidney HEK293T cells were cultured in DMEM medium(Thermo Scientific) containing 10% fetal bovine serum. All cell lines were cultured in a medium with 100 U/mL penicillin and 100 μg/mL streptomycin (Gibco) at 37°C in a humidified 5% CO_2_-containing atmosphere.

### Nanobody screening

Total RNA was isolated from peripheral blood lymphocytes obtained from *Camelus dromedarius* immunized with CD7-positive Jurkat cells using TRIzol reagent (Invitrogen), used as a template for cDNA synthesis (Fermentas), and cloned into the phagemid vector pComb3XSS. Propagation of VHH libraries and filamentous phages was performed by following published procedure [51]. Panning of phage display libraries with intact cells was carried out as described previously [36] using 293T and CD7-expressed 293T cell lines. After four rounds of screening, 30 clones were selected to characterize their cell binding using a whole-cell enzyme-linked immunosorbent assay as described previously [36], in which CD7-negative and -positive 293T cells were used as controls. All CD7-positive and some CD7-negative nanobody clones were selected for sequencing.

### Production and purification of CD7-specific nanobodies

To induce soluble expression of nanobody fragments, two cDNAs coding for CD7-specific nanobodies were subcloned into the expression vector pET28a containing 6×His and HA tags, which allowed for purification and characterization of the nanobodies. The plasmids were propagated in *E.coli* BL21 (DE3). Induction of expression and purification of the two CD7- specific nanobodies was performed as follows. A *E.coli* BL21 (DE3)-transformed plasmid was cultured in 3 mL of LB broth in the presence of 100 μg/mL kanamycin at 37°C overnight. The culture was then diluted at 1:100 in fresh LB medium containing 100 μg/mL kanamycin. At the early log phase (opitical density at 600 nm = 0.6–0.8) of bacterial growth, protein expression was induced at 22°C by adding 0.5 mM isopropyl β-D-1-thiogalactopyranoside (IPTG, Sangon Biotech) to the culture for 6–8 hours. The nanobodies were then purified using Ni-NTA agarose (GE Healthcare Life Sciences) according to the manufacturer's protocol. Imidazole was removed using dialysis with phosphate-buffered saline (PBS) overnight at 4°C. Protein fractions and purity were analyzed via sodium dodecyl sulfate-polyacrylamide gel electrophoresis. Protein concentrations were determined using a BCA assay (Beyotime). One CD7 negative nanobody was purified as negative control.

### Construction and expression of nanobody-based immunotoxins

Sequence coding for the CD7-specific nanobody (VHH6) and then truncated ETA were synthesized with an N-terminal 6-His tag and a 20-amino-acid linker 4(G_4_S) between the nanobody and truncated ETA. The C-terminal REDLK retention motif against the KDEL motif was substituted with the KDEL sequence. The sequence was then ligated in the expression vector pET28a, which was then named pET28a-VHH6-PE38-KDEL. To prepare a bivalent nanobody-based immunotoxin, another VHH6 sequence was inserted into pET28a-VHH6-PE38-KDEL, and the resulting sequence was named pET28a-dVHH6-PE38-KDEL. Expression of the nanobody-based immunotoxins was induced at low temperatures as described above, and the immunotoxins were named PG001 (monovalent nanobody immunotoxin; VHH6-PE38) and PG002 (bivalent nanobody immunotoxin; dVHH6-PE38). The immunotoxins were then purified as described above. In addition, one CD7 negative nanobody of VHH22 was prepared and constructed as irrelevant immunotoxins in monovalent and bivalent forms.

### Determination of the cell-binding ability and affinity of nanobodies, PG001 and PG002

To evaluate the cell-binding ability of the nanobodies and immunotoxins, nanobodies or immunotoxins at different concentrations were incubated with Jurkat cells on ice. After two washes with ice-cold PBS, a bound nanobody or immunotoixn was detected with an Allophycocyanin(APC)-labeled anti-HA tag antibody(Miltenyi Biotec) or an Alexa Fluor 488-labeled anti-His tag antibody(Qiagen) in PBS containing 2% bovine serum albumin for 1 hour on ice. Cells were then washed twice with PBS, and their fluorescence was measured using a FACS Calibur (BD Biosciences). Binding saturation curves, and Scatchard plots were generated and nonlinear regression analysis was performed using the Prism software program (Graph Pad Software).

### Flow cytometric analysis

The binding of nanobodies to cells (jurkat, CEM, RPMI8226, H460, 293T and Ramos cells) was analyzed using a FACS Calibur (BD Biosciences). Cells were stained with nanobodies as described previously [37]. Ten thousand events were collected for each cell sample, and analyses of whole cells were performed using appropriate scatter gates to exclude cellular debris and aggregates. To monitor binding of nanobodies and immunotoxins, cancer cells were incubated with immunotoxins at a concentration of 5 μg/mL, after two washes and then add an Allophycocyanin(APC)-labeled anti-HA tag antibody(Miltenyi Biotec) or an Alexa Fluor 488-labeled anti-His tag antibody(Qiagen) for one hour. After a final wash, the cells were analyzed using fluorescence-activated cell sorting. The CD7 expression in patient derived leukemia cells was used the commercially available anti-human CD7 IgG antibody labeled with phycoerythrin (PE) (BD Pharmingen^™^) to test.

### Cell immunofluorescence

To determine the specificity of the nanobodies, a cell immunofluorescence assay was performed. Jurkat and RPMI8226 cells were seeded in a six-well plate containing coverslips treated with poly-L-lysine (Beyotime), and incubated at 37°C overnight. The cells were washed with ice-cold PBS three times and fixed in 4% paraformaldehyde, the fixative was subsequently quenched with 100 mM glycine in PBS for 15 minutes. Nanobodies were added to fixative at 5 μg/mL for 45 minutes. Bound nanobodies were detected using a rabbit-anti-HA tag (1:1000) in PBS containing 2% bovine serum albumin followed by Alexa Fluor 488-conjugated anti-rabbit IgG for 1 hour at room temperature for each reaction. Cells were washed twice with PBS containing 2% bovine serum albumin, and the cells fluorescence was measured using confocal fluorescence microscopy. In addition, H460 cells were transfected with the empty vector pcDNA3.1 and pcDNA3.1-CD7 using lipofectamine 2000. The CD7 expression on transfected H460 cells was then detected using a nanobody with immunofluorescence as described above. At the same time, the CD7 expression on transfected H460 cells was assessed using a commercially available anti-human CD7 IgG antibody labeled with phycoerythrin(PE) (BD Pharmingen^™^) and nanobody VHH6 as described above.

### Immunotoxins cytotoxicity assay

Survival of cells treated with immunotoxins was measured using a WST-8 assay with a Cell Counting Kit-8 (Dojindo Molecular Technologies). Briefly, 1 × 10^4^ cells per well in a 96-well plate were incubated with immunotoxins at various concentrations (H460 and 293 T cells were seeded at 3 × 10^3^/well) for 72 hours, after which 10 μL of CCK-8 reagent was added to the wells. The plate was incubated until the wells with a maximum absorbance at 450 nm. To determine whether cell-growth inhibition was attributable to apoptosis, cells were seeded in 24-well plate at 2.5 × 10^**5**^/mL and treated with the immunotoxins. Whole cells were stained with fluorescein isothiocyanate-conjugated annexin V and 7-amino-actinomycin D (7-AAD;BD Pharmingen^™^) according to the manufacturer's protocol. For experiments with primary samples obtained from patients, cells were seeded in 24- well plates at 4 × 10^**5**^/mL and treated with 500 ng/mL PG001 and 100 ng/mL PG002. For blocking experiments, cells were seeded at 2.5 × 10^**5**^/mL(cell line) or 4 × 10^**5**^/mL (primary cells) in 24-well plates, and a 100-fold molar excess of the parental nanobody or irrelevant nanobody was added to the culture 1 hour before adding the immunotoxin.

### SDS gel electrophoresis and Western blot analysis

Gels were stained with Coomassie brilliant blue R250 (Amresco). Also, Western blots with secondary antibodies coupled with horseradish peroxidase were performed. Immobilon Western Chemiluminescent HRP Substrate (Merck Millipore) was used for detection. PG001 was detected using an anti-His tag rabbit antibody (CST). Full-length poly-(ADP-ribose) polymerase (PARP) and its specific cleavage products were detected using a mouse anti-human PARP antibody (Beyotime).

### Size exclusion chromatography analysis

Size exclusion chromatography (SEC) of VHH6, PG001, and PG002 was performed by Superdex 200^™^ (GE Healthcare). Superdex separations were carried out in PBS. Molecular markers ribonuclease A (13.7 kDa), BSA (66 kDa) and aldolase (158 kDa) were utilized as the calibration standards.

### Inhibition of leukemia cell engraftment in NOD/SCID mice

100 μL of 2 × 10^6^ CEM cells were injected intravenously into the lateral tail vein of NOD/SCID mice (Shanghai SLAC Laboratory Animal Co. Ltd) on day 0 of the experiment. Mice were then given four intravenous injections of 5 μg of PG001 and PG002 and monitored daily. Moribund mice were killed according to experimental animal management regulations. Survival durations were recorded for evaluation of therapeutic efficacy using Kaplan-Meier analyses, and the median survival duration was determined.

### Statistical analysis

Survival comparisons among groups were analyzed by log-rank test (GraphPad Prism 5). Student's *t*-test was used. *P*-values below 0.05 were considered statistically significant. Correlation analyses were carried out using the correlation test with a confidence interval of 95%.

## SUPPLEMENTARY MATERIALS FIGURES


