# Supplementary material for: Novel CD7-specific nanobody-based immunotoxins potently enhanced apoptosis of CD7-positive malignant cells
Source: Oncotarget. 2016 Apr 12;7(23):34070–83. doi: 10.18632/oncotarget.8710 (PMC5085138; doi:10.18632/oncotarget.8710)
Supplement: Supplementary file 1 [file oncotarget-07-34070-s001.pdf]

Novel CD7-specific nanobody-based immunotoxins potently enhanced apoptosis of CD7-positive malignant cells

Supplementary Materials

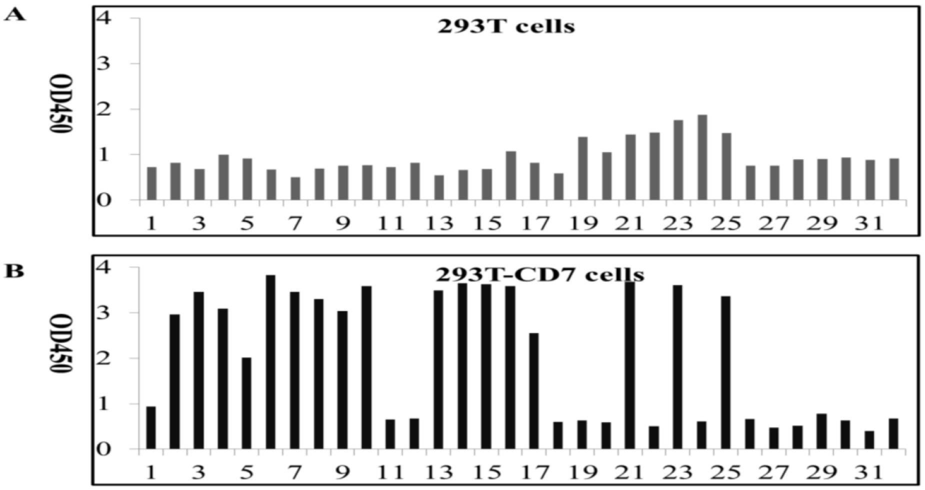

Supplementary Figure S1: Whole-cell enzyme-linked immunosorbent assay results for phage nanobodies prepared from 30 clones picked after four rounds of selection with 293T-CD7 cells. The phage nanobodies were screened using CD7-negative 293T cells (**A**) and 293T-CD7 cells (**B**). The last two wild-type phages were used as negative controls.

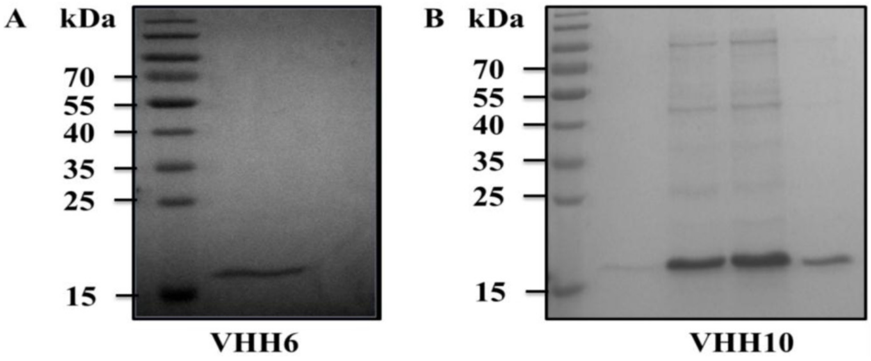

Supplementary Figure S2: Purity of the purified the nanobodies. The two nanobodies, VHH6 (**A**) and VHH10 (**B**), were expressed in *E. coli* BL21 (DE3), purified via affinity chromatography, and evaluated using Coomassie brilliant blue staining.

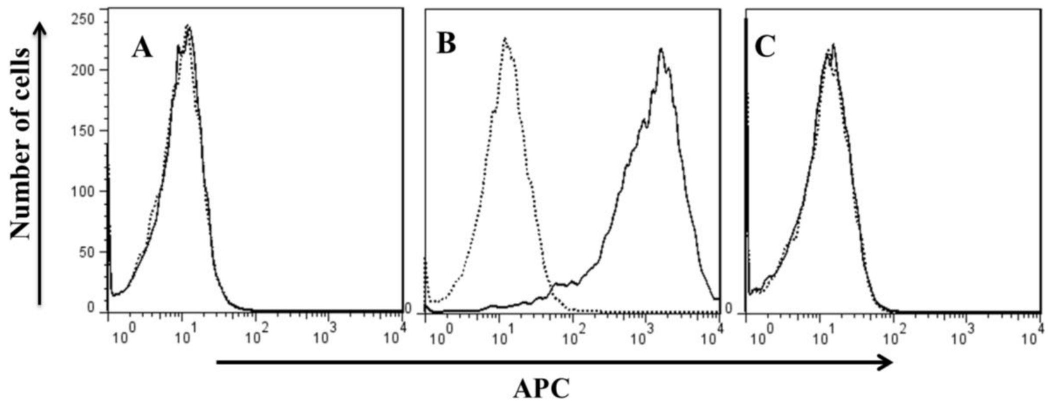

Supplementary Figure S3: The specificity of VHH6 on CD7-positive cells. The cells were stained with purified VHH6 and examined using flow cytometric analysis. Cells were stained with purified nanobody VHH6 (solid line) or with a irrelevant nanobody (dotted line) at the same concentration and analyzed by FACS. (**A**) CD7 negative 293T cells. (**B**) 293T-CD7 cells. (**C**) CD7 negative Ramos cells.

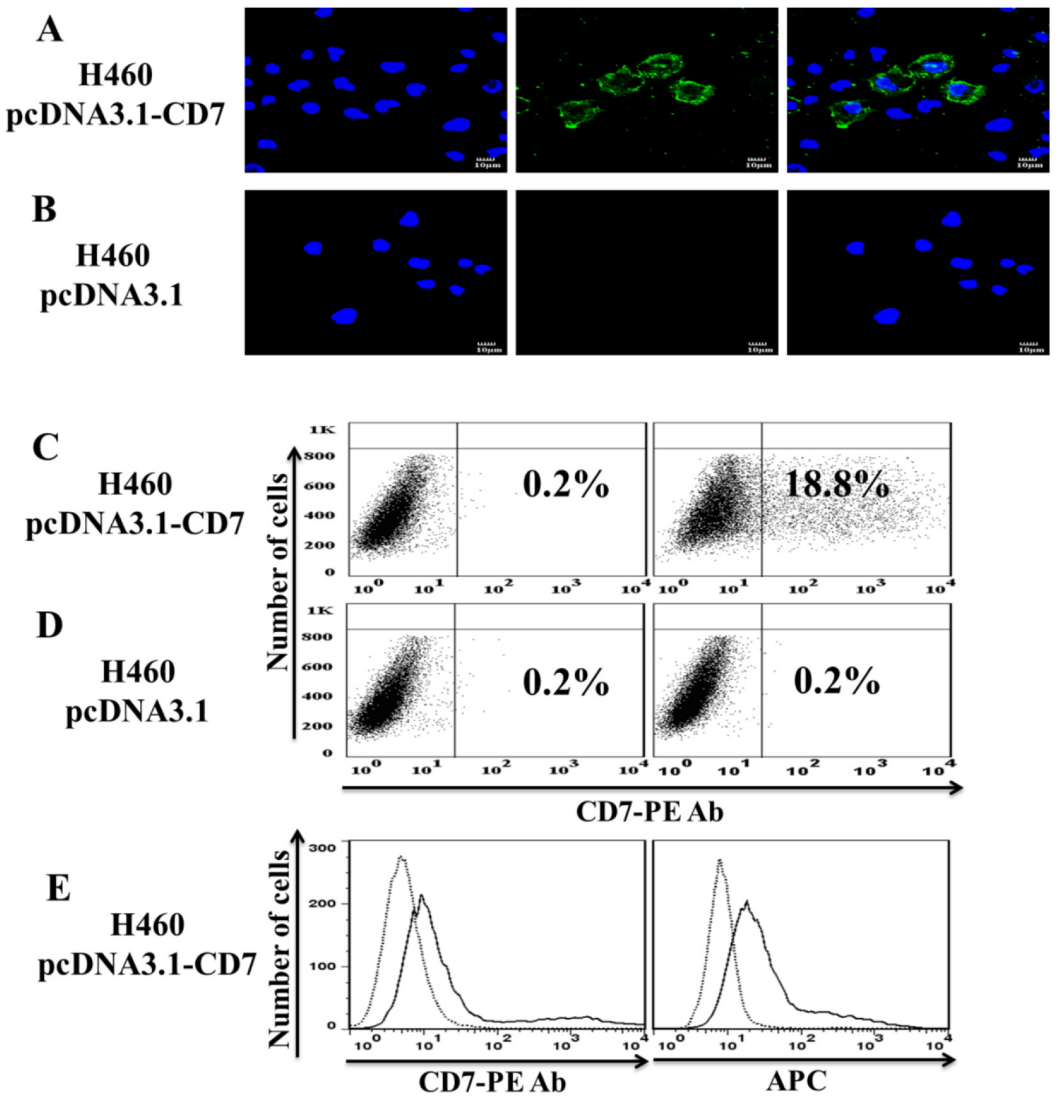

Supplementary Figure S4: Expression of CD7 in H460 cells as measured using VHH6 and commercial antibodies. H460 cells were transfected with pcDNA3.1-CD7 or the empty vector pcDNA3.1 using Lipofectamine 2000. The CD7 expression in transfected H460 cells was detected using confocal fluorescence microscopic analysis with VHH6 (**A** and **B**) and flow cytometric analysis with commercial antibody (**C** and **D**). (**E**) H460 cells trasfected with pcDNA3.1-CD7. The CD7 expression of H460 cells were stained with commercial antibody (left) or with nanobody VHH6 (right) and analyzed by FACS, dotted line as the isotype control. The data are representative of three separate experiments.

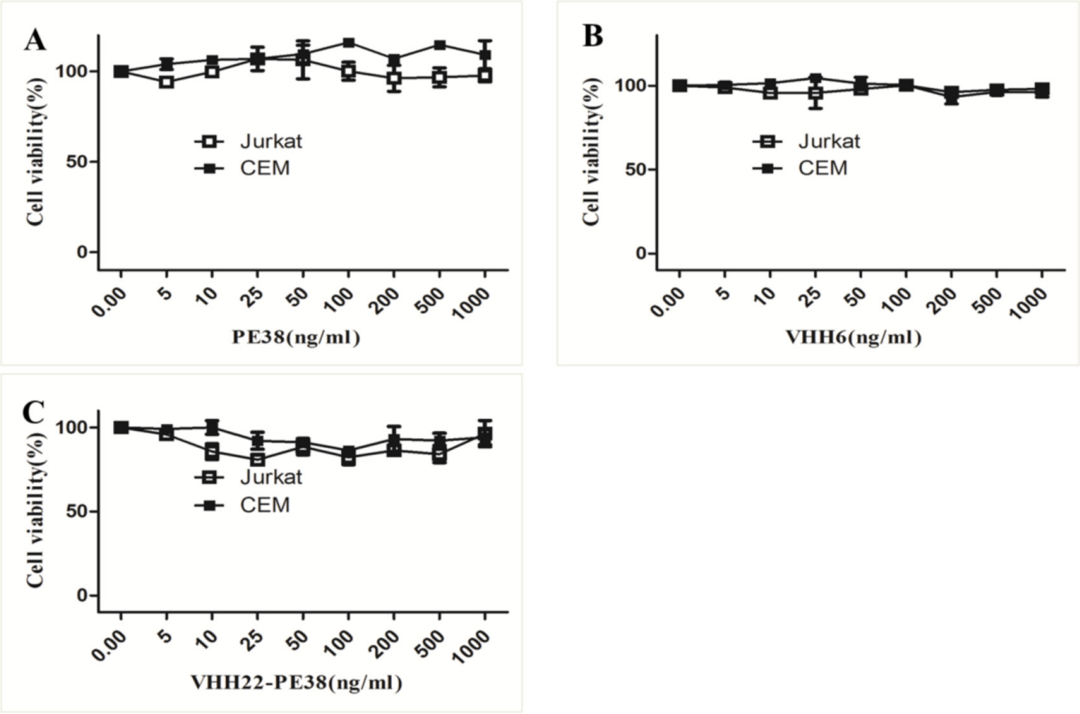

Supplementary Figure S5: The effect of VHH6 and PE38 on the viabilities of Jurkat and CEM cells. CD7-positive Jurkat cells and CEM cells were treated withVHH6 (**A**), PE38 (**B**) and VHH22-PE38 (**C**) at different concentrations for 72 hours. Cell-growth inhibition was measured using a WST-8 assay. The bars represent the mean values from three independent experiments. Standard deviations are indicated by the error bars.

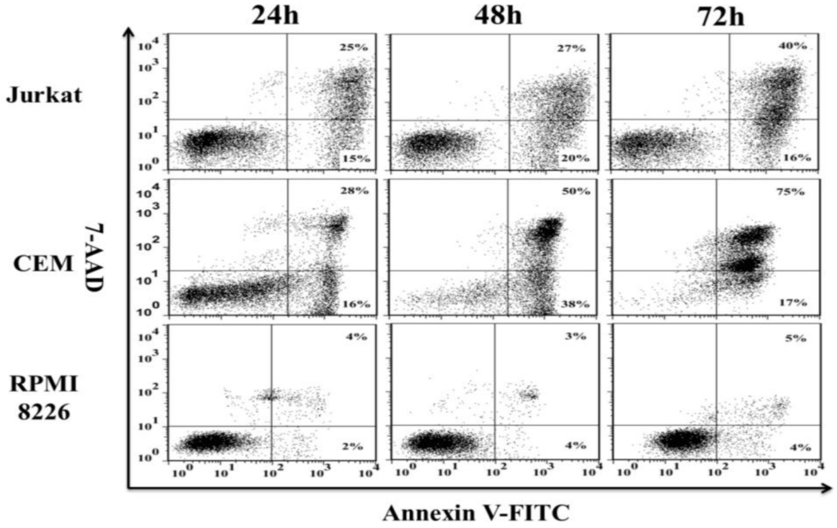

Supplementary Figure S6: Induction of leukemic T-cell growth inhibition by PG001 via apoptosis in a time-dependent manner. Jurkat, CEM, and RPMI8226 cells were treated with a single dose 150 ng/mL PG001 and then stained with annexin V and 7-AAD at the indicated time points. The percentage of cells undergoing early apoptosis (annexin V-positive and 7-AAD-negative) is shown in the bottom right quadrant of each plot. The percentage of dead cells (annexin V- and 7-AAD-positive) shown in the upper right quadrant of each plot. The data are representative of three separate experiments.

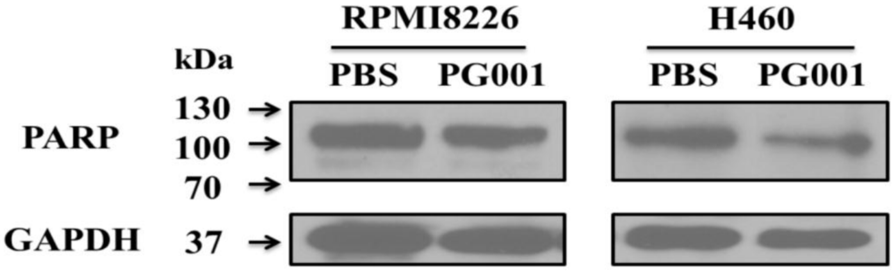

Supplementary Figure S7: Failure of PG001 to induce cleavage of PARP in CD7-negative RPMI8226 and H460 cells. Western blots of PARP cleavage in CD7-negative RPMI8226 and H460 cells that were left untreated (PBS) or treated with 150 ng/mL PG001 are shown.

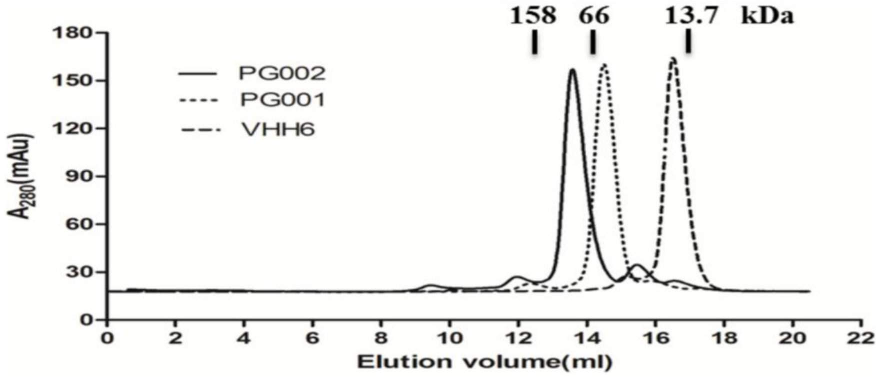

Supplementary Figure S8: Characterization of VHH6, PG001, and PG002 with size exclusion chromatography. Purified VHH6, PG001, and PG002 were performed size exclusion chromatography by using a Superdex200™ column. Superdex separations were carried out in PBS. The elution positions of molecular mass markers are indicated.

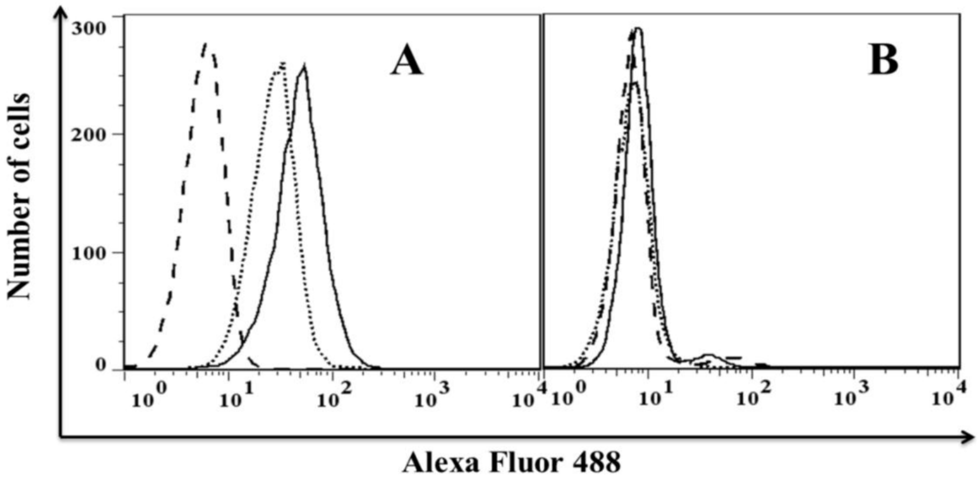

Supplementary Figure S9: Binding ability assay of PG001 and PG002 in the Jurkat and H460 cells. Cells were stained with purified PG002 (solid line), PG001 (dotted line) or an irrelevant bivalent immunotoxin (dashed line) and analyzed using flow cytometry. (**A**) CD7-positive Jurkat cells. (**B**) CD7-negative H460 cells. The data are representative of three separate experiments.

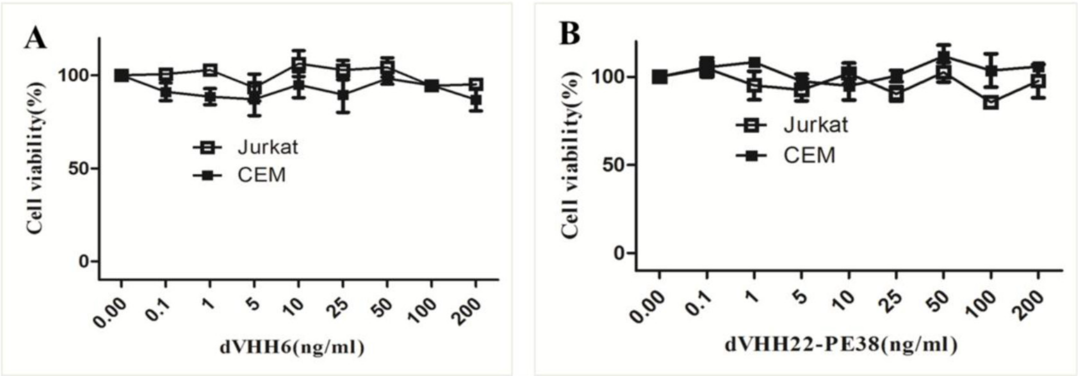

Supplementary Figure S10: The effect of dVHH6 and dVHH22-PE38 on the viabilities of Jurkat and CEM cells. CD7-positive Jurkat cells and CEM cells were treated with dVHH6 (**A**) and dVHH22-PE38 (**B**) at different concentrations for 72 hours. Cell-growth inhibition was measured using a WST-8 assay. The bars represent the mean values from three independent experiments. Standard deviations are indicated by the error bars.

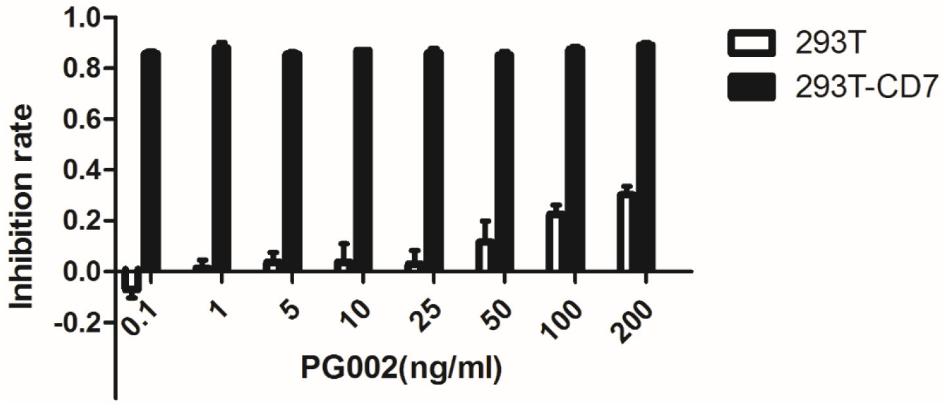

Supplementary Figure S11: Specific induction of 293T-CD7 and 293T cells by PG002 in a dose-dependent manner. CD7-positive 293T-CD7 cells and CD7-negative 293T cells were treated with PG002 at different concentrations for 72 hours. Cell-growth inhibition was measured using a WST-8 assay. The bars represent the mean values from three independent experiments. Standard deviations are indicated by the error bars.

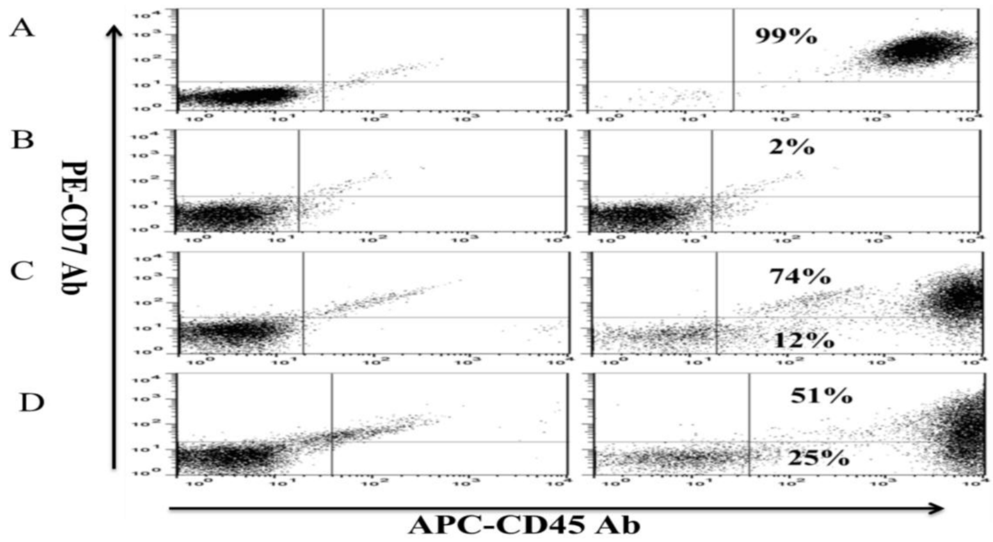

Supplementary Figure S12: CD7 detection of CEM cells before and after injection in NOD/SCID mice. (**A**) *In vitro* cultured CEM cells; (**B**) Bone marrow cell suspensions obtained from a naïve NOD/SCID mouse; (**C**) spleen and (**D**) bone marrow cell suspensions obtained from a NOD/SCID mouse treated with PG002. Those cells were analyzed using fluorescence-activated cell sorting with the anti-human CD45-APC and anti-human CD7-PE antibodies (right panel), or PE-/APC-labeled isotype antibody controls (left panel).
